# Binding of micro-nutrients to the cell wall of the fungus *Schizophyllum commune*

**DOI:** 10.1016/j.tcsw.2023.100108

**Published:** 2023-06-17

**Authors:** Fleur E.L. Kleijburg, Adil A. Safeer, Marc Baldus, Han A.B. Wösten

**Affiliations:** a Microbiology, Department of Biology, Utrecht University, Padualaan 8, 3584 CH Utrecht, The Netherlands; b NMR Spectroscopy, Bijvoet Center for Biomolecular Research, Utrecht University, Padualaan 8, 3584 CH Utrecht, The Netherlands

**Keywords:** Fungus, *Schizophyllum commune*, Cell wall, Micronutrients, Sorption

## Abstract

The cell wall fulfils several functions in the biology of fungi. For instance, it provides mechanical strength, interacts with the (a)biotic environment, and acts as a molecular sieve. Recently, it was shown that proteins and β-glucans in the cell wall of *Schizophyllum commune* bind Cu^2+^. We here show that the cell wall of this mushroom forming fungus also binds other (micro-)nutrients. Ca^2+^, Mg^2+^, Mn^2+^, NO_3_^–^, PO_4_^3-^, and SO_4_^2-^ bound at levels > 1 mg per gram dry weight cell wall, while binding of BO_3_^-^, Cu^2+^, Zn^2+^ and MoO_4_^2-^ was lower. Sorption of Ca^2+^, Mn^2+^, Zn^2+^ and PO_4_^3-^ was promoted at alkaline pH. These compounds as well as BO_3_^3-^, Cu^2+^, Mg^2+^, NO_3_^–^, and SO_4_^2-^ that had bound at pH 4, 6, or 8 could be released from the cell wall at pH 4 with a maximum efficiency of 46–93 %. Solid-state NMR spectroscopy showed that the metals had the same binding sites as Cu^2+^ when a low concentration of this ion is used. Moreover, data indicate that anions bind to the cell wall as well as to the metal ions. Together, it is shown that the cell wall of *S. commune* binds various (micro-)nutrients and that this binding is higher than the uptake by hyphae. The binding to the cell wall may be used as a storage mechanism or may reduce availability of these molecules to competitors or prevent toxic influx in the cytoplasm.

## Highlights

• Micro-nutrients bind to the cell wall of Schizophyllum commune.
• Binding of micro-nutrients to the cell wall is reversible.
• Release of micro-nutrients from the cell wall is promoted at low pH.
• Cations bind to cell wall proteins.
• Anions bind to the cell wall as well as to the cations within the cell wall.

## 1 Introduction

Fungi are essential in nature for nutrient recycling and by establishing pathogenic and mutual beneficial interactions with plants and animals. Cell walls can make up 30% of the volume of yeast cells and hyphae (Garcia et al., 2020). This outer layer plays an important role in the interaction with the (a)biotic environment, provides mechanical strength and acts as a molecular sieve. Traditionally, fungal cell wall composition has been studied by enzymatic and/or chemical treatments. This revealed that (glyco)proteins, glucan, and chitin are the main constituents of the cell wall (Gow et al., 2017, Garcia-Rubio et al., 2020). Recently, solid state NMR (ssNMR) was used to study the cell wall of the saprotrophic fungi *Schizophyllum commune* (Ehren et al., 2020, Safeer et al., 2023) and *Aspergillus fumigatus* (Kang et al., 2018, Chakraborty et al., 2021). This non-destructive method revealed that the cell wall of *S. commune* contains a rigid core of α- and β-(1,3)-glucan, β-(1,3)-(1,6)-glucan, highly branched and single stranded β-(1,4)-chitin as well as polymeric fucose and mannose. The mobile fraction of the cell wall is composed of β-(1,3)-glucan, β-(1,3)-(1,6)-glucan, β-(1,6)-glucan, as well as polymeric mannose and polysaccharides containing N-acetyl galactosamine (GalNAc) and its deacetylated variant (GalN) (Ehren et al., 2020, Safeer et al., 2023). The α-(1,3)-glucan is the most dominant polysaccharide in the rigid core of the cell wall, while short β-glucan chains are most dominant in the mobile layer together with longer β-(1,3)(1,6)-glucan chains. Solid state NMR also revealed that the cell wall contains proteins and lipids (Ehren et al., 2020, Safeer et al., 2023). Their nature has not been revealed yet except for the SC3 hydrophobin (Safeer et al., 2023). KOH extraction removes part of the cell wall molecules, including the lipids and protein, but still contains both mobile and rigid molecules (Ehren et al., 2020). The mobile fraction consists of β-(1,3)-(1,6)-glucan, while the rigid part also consists of this molecule as well as β-(1,3)-glucan, chitin, α-(1,3)-glucan and polymeric fucose.

Interaction of fungi with high concentrations of metals has been widely studied. For instance, *S. commune* grows about 8 mm per day inside the Chernobyl exclusion zone in soil contaminated with radioactive heavy metals (Traxler et al., 2021). It tolerates > 10 mg l^-1^Cd, > 100 mg l^−1^ Zn and U, and > 1 g l^−1^ Ca, Fe, Sr and Cs (Merten et al., 2004, Günther et al., 2014, Traxler et al., 2022). A total of 55% and 65% Fe and Ca had been sorbed by *S. commune* when this fungus had been incubated in seepage water from a former uranium mining site that contained 2.5 and 1.3 g l^−1^ of these elements, respectively (Merten et al., 2004). Cu and U were found to bind to the cell wall (Günther et al., 2014, Safeer et al., 2023). At low concentration (47 mg l^−1^), Cu^2+^ mainly binds to proteins in the mobile fraction of the cell wall, while some binding is observed to polysaccharides (Safeer et al., 2023). On the other hand, Cu^2+^ binds to both proteins and the C5 of rigid (1,3)(1,6)-β-glucan when exposed to a high concentration of the metal (>1 g l^−1^). Metals can also be taken up by hyphae (Günther et al., 2014, Kirtzel et al., 2019, Traxler et al., 2022). It was shown that Cd, Cs, Sr, and Zn can be transported within the mycelium. In the case of Cs and Sr this can be for tens to hundreds of cellular compartments away from the metal source (Traxler et al., 2022).

Here, we studied sorption of cations (including metals) and anions to the mycelium of *S. commune* at concentrations found in trace element solutions used for growing organisms. We show that the cell wall of *S. commune* not only binds Cu^2+^ but also other metals (Ca^2+^, Mg^2+^, Mn^2+^ and Zn^2+^) as well as anions (NO_3_^–^, PO_4_^3-^, SO_4_^2-^, BO_3_^-^, and MoO_4_^2-^). Solid-state NMR showed that the metals have the same binding sites as Cu^2+^. Our results also indicate that anions bind to the cell wall directly as well as to metals that have been sorbed by the cell wall. Moreover, results show that uptake by the hyphae represents a minor fraction of the total sorption of micro-nutrients by the mycelium.

## 2 Materials and methods

### 2.1 Strain and culture conditions

*S*. *commune* strain H4-8A (matA_43_matB_41_; FGSC no. 9210) (Ohm et al., 2010) was grown at 30 °C on minimal medium with or without 15 g l^−1^ agar (Dons et al., 1979) with the modification that L-asparagine was replaced by 1.32 g l^−1^ (NH_4_)_2_SO_4_ (called MM-NA and MM-N, respectively) (Table 1). A quarter of a 7-day-old colony grown on MM-NA was macerated in 50 ml MM-N for 30 sec at 18000 rpm in a Waring 2 Speed Blender (Waring Laboratory Science, https://www.waringlab.com). The homogenate was incubated for 24 h at 200 rpm in a 250 ml Erlenmeyer. The culture was macerated again (see above) and 200 mg wet-weight mycelium was grown in 100 ml MM-N in a 250 ml Erlenmeyer for 7 days at 200 rpm. Alternatively, 40 mg wet weight mycelium was grown in a 100 ml Erlenmeyer in 20 ml MM-N containing ^13^C-glucose and ^15^NH_4_. Mycelium was harvested by centrifugation for 10 min at 10,000 g.

**Table 1:** **Micro-nutrient composition of the medium and the micro-nutrient and anion solutions that were used in the study**. Living mycelium and cell wall extracts were incubated in the latter two solutions to assess binding of anions and cations to the cell wall (living mycelium and cell wall extracts) and / or their uptake by the hyphae (living mycelium).

Micronutrient	Minimal Medium (mg l^−1^)	Micro-nutrient solution (mg l^−1^)	Anion solution (mg l^−1^)
BO_3_^3-^	0.06	4.12	4.12
Ca^2+^	0.2	80.16	NA
Cl^-^	2.09	67.36	NA
Co^2+^	0.1	NA	NA
Cu^2+^	0.05	1.59	NA
Fe^3+^	1.03	NA	NA
K^+^	581.05	293.23	NA
Mg^2+^	49.3	72.92	NA
Mn^2+^	0.02	38.46	NA
MoO_4_^2-^	0.05	2.06	2.06
Na^+^	NA	11.95	NA
NH_4_^+^	360.42	NA	318.4
NO^3–^	0.63	558.04	558.04
PO_4_^3-^	866.21	237.43	237.43
SO_4_^2-^	1.16	295.38	295.38
Zn^2+^	0.45	3.27	NA

### 2.2 Isolation of mycelium and cell walls

Mycelium, either or not ^13^C^15^N-labeled (see above), was washed three times with 80 ml or 40 ml of demineralized water (dH_2_O), respectively, each wash followed with a 10-min centrifugation at 10,000 g. This resulted in the non-labelled and labelled living mycelium fractions that were used in binding studies. In addition, part of these living mycelium fraction were freeze-dried and homogenized with five metal beads for 9 min in an SK550 1.1 heavy-duty paint shaker (Fast & Fluid, https://www.fast-fluid.com). The resulting homogenates were washed four times with 240 ml or 120 ml dH_2_O and centrifuged (see above) between the washes. This resulted in the non-labelled and labelled water-extracted cell wall fractions. Half of the non-labelled water-extracted cell wall fraction was taken up in 120 ml 1 M KOH and incubated at 60 °C for 20 min with manual shaking every 5 min. A volume of 11.4 ml glacial acetic acid was added, and the suspension was centrifuged (see above). Incubation in 1 M KOH, precipitation with glacial acetic acid and centrifugation were repeated using the precipitate. The cell walls were washed twice with 120 ml dH_2_O, each time followed by centrifugation (see above), resulting in the KOH-extracted cell wall fraction.

### 2.3 Sorption and desorption of micronutrients to and from mycelium and cell walls

The water- and KOH-extracted cell walls made up 24% ± 0.06 and 13% ± 0.02 of the mycelium, respectively. Therefore, aliquots of 1 g wet-weight living mycelium, 0.24 g wet-weight water-extracted cell walls and 0.13 g wet-weight KOH-extracted cell walls were suspended in 10 ml water, 10 ml micro-nutrient solution, or 10 ml anion solution (Table 1). Samples were incubated at room temperature for 30 min at 10 rpm on a L29 Test-Tube Rotator (Labinco BV, https://www.labinco-bv.com). The pH of the suspension was set at 4, 6, or 8 with 0.1 M HCl or 0.1 M NaOH, and the incubation was prolonged for 30 min. The suspensions were filtered over Miracloth (Merck Millipore, https://www.merckmillipore.com) and the supernatant was collected. A total of 5 mg wet weight aliquot of ^13^C^15^N-labeled water-extracted cell wall was suspended in 1 ml micro-nutrient solution (with 2.79 mg l^−1^ Fe^3+^) or anion solution at pH 5.5.

To assess desorption of micro-nutrients, living mycelium and cell wall extracts were washed two times with 12 ml 1 µM NaOH (pH 8), after which samples were incubated in 10 ml 0.1 mM HCl (pH 4) for 30 min at 10 rpm on a Test-Tube Rotator (see above) and the supernatant collected.

### 2.4 Micro-nutrient quantification and data analyses

Micro-nutrient solutions were filtered over a 0.2 µm Filtropur S microfilter (Sarstedt Inc., https://www.sarstedt.com). The concentration of NO_3_^–^ and NH_4_^+^ was determined on a SAN^++^ System Continuous Flow Analyzer (CFA)(Skalar, https://www.skalar.com) using the cadmium reduction reaction and the Berthelot reaction, respectively. The system sample and wash times were set at 80 sec. A dilution series of the original anion solution was used as a calibration curve. Measurements were corrected by subtracting the light absorption recorded for a dH_2_0 blank. The amounts of NO_3_^–^ and NH_4_^+^ in the solution were calculated with linear regression models. The concentration of BO_3_^3-^, Ca^2+^, Cu^2+^, Mg^2+^, Mn^2+^, MoO_4_^2-^, PO4^3-^, SO4^2-^ and Zn^2+^ were determined on an iCAP 6000 Series Inductively Coupled Plasma Optical Emission Spectrometer (ICP-OES)(ThermoFischer Scientific, https://www.thermofisher.com). To this end, undiluted supernatant was acidified with nitric acid (1% w/v). The counts per second of BO_3_^2-^, Ca^2+^, Cu^2+^, Mg^2+^, Mn^2+^, Mo^6+^, PO_4_^3-^, SO_4_^2-^, and Zn^2+^ were recorded at 249.773 nm, 422.673 nm, 327.396 nm, 279.079 nm, 293.930 nm, 202.030 nm, 214.914 nm, 182.034 nm, and 206.200 nm, respectively. A dilution series of the original micro-nutrient solution was used as a calibration curve. Measurements were corrected by subtracting the counts per sec recorded for a dH2O blank. The amounts of each micronutrient in the solution was calculated with linear regression models.

### 2.5 Solid-state NMR

Binding of micro-nutrients to the water-extracted ^13^C^15^N-labeled cell wall fraction was assessed via fast magic angle spinning (MAS) solid state (ss) NMR. Spectra were acquired on a narrow bore 16.5 T (700 MHz) NMR spectrometer with a 1.3 mm HXY MAS probe (Bruker BioSpin). Spectra of micronutrient solution-bound and anionic-solution bound *S*. *commune* cell wall samples, were recorded at set temperatures of 258 K and 255 K and MAS frequencies of 60 kHz and 55 kHz, respectively. The actual sample temperatures were estimated to be 298 K due to frictional heating, as previously calibrated using a KBr powder sample (Thurber and Tycko, 2009). To probe the flexible cell wall components that bind ions (Safeer et al., 2023), scalar-based ^1^H–^13^C correlation with WALTZ16 (Shaka et al., 1983) decoupling on the ^1^H and ^13^C channels was performed during the experiments (Bax et al., 1990). The ^13^C offset was set at 51 ppm with a spectral width of 130 ppm to sample NMR signals from both polysaccharides to amino acid side chains. Rigid components were probed using dipolar-based ^1^H–^13^C two-dimensional correlation spectra using 70–100% forward and backward cross-polarization (CP) ramps with, respectively, 1.2 ms and 0.2 ms forward and backward CP contact times. PISARRO (Weingarth et al., 2009) decoupling was applied on both ^1^H and ^13^C channels during the dipolar-based experiments and the ^13^C offset was set at 57 ppm with a spectral width of 130 ppm. For all experiments proton and carbon 90° pulses were applied at 156 kHz and 78 kHz, respectively and water suppression was achieved by applying MISSISSIPPI at 23 kHz. Proton chemical shifts were referenced to the water peak at 4.7 ppm and ^13^C chemical shifts were referenced to data published by Ehren et al. (2020). NMR data was analyzed using TopSpin 4.1 (Bruker BioSpin).

### 2.6 Statistics

Sorption and release of cations and anions were analyzed using two-way and three-way ANOVA, respectively, with TukeyHSD post hoc test (p ≤ 0.05). Differences in sorption and release of anions from micro-nutrient or anion solution were analyzed using t-tests (p ≤ 0.05).

## 3 Results

### 3.1 Binding of ions to the *S. commune* cell wall

*S. commune* was grown for 7 days in a defined medium (Table 1). Water content of the mycelium was 92% ± 0.01. The dry weight water- and KOH-extracted cell wall made up 24% ± 0.06 and 13% ± 0.02 of the dry weight mycelium, respectively. Therefore, 1 g wet weight living mycelium, 0.24 g wet weight water-extracted cell walls and 0.13 g wet weight KOH-extracted cell walls were incubated in 10 ml micro-nutrient or anion solution (Table 1) at pH 4, 6 and 8. The reduction of the concentration of micro-nutrients in these solutions (that are based on the Hoagland solution used to grow plants) was determined to deduce binding of the micro-nutrients to the cell wall and, in the case of the living mycelium, uptake within the mycelium as well. In the next sections only significant differences between the living mycelium and the cell wall extracts and the different pH conditions are described.

The release of micro-nutrients from the mycelium or cell walls in the control (i.e. incubation in water at pH 4, 6, and 8 in the absence of micro-nutrients or anions) (Table 2) was first assessed. These values were taken into account to determine the amount of micro-nutrients that had been bound or taken up when incubated in the micro-nutrient or anion solution. The amount of ions released from the living mycelium or the water- and KOH-extracted cell walls did not depend on the pH of the water. Most micro-nutrients were released from the water-extracted cell walls, ranging from 0.19 µg (NH_4_^+^) to 254.00 µg (PO_4_^3-^). Release from the living mycelium was between 0.00 µg (BO_3_^-^) and 83.54 µg (PO_4_^3-^), while it ranged between 0.00 µg (Mn^2+^ and Zn^2+^) and 81.59 µg (NO_3_^–^) in the case of the KOH-extracted cell walls.

**Table 2:** **Release of micro-nutrients from the mycelium or cell walls at different pH values**. Release (µg ± sd) from 1 g wet weight living mycelium, 0.24 g wet weight water-extracted cell walls and 0.13 g wet weight KOH-extracted cell walls was determined after 30 min incubation in dH_2_O at pH 4, 6, or 8.

Micro-nutrient	Living mycelium			dH20-extracted cell walls			KOH-extracted cell walls		
	pH 4	pH 6	pH 8	pH 4	pH 6	pH 8	pH 4	pH 6	pH 8
BO_3_^3-^	0.00	0.00	0.00	2.34	2.89	2.89	0.42	0.40	0.44
	±0.00	±0.00	±0.00	±4.06	±5.01	±5.00	±3.94	±0.70	±0.77
Ca^2+^	0.19	0.16	0.21	13.83	31.12	81.67	0.44	0.38	0.33
	±0.11	±0.17	±0.21	±23.05	±53.11	±140.74	±0.37	±0.33	±0.31
Cu^2+^	0.11	0.12	0.14	1.18	3.48	4.13	3.09	0.16	0.15
	±0.17	±0.19	±0.24	±1.65	±5.62	±6.78	±0.23	±0.27	±0.25
Mg^2+^	3.46	1.23	2.29	13.12	26.33	40.42	0.06	0.00	0.02
	±1.36	±0,75	±2,09	±19,75	±44,18	±68,78	±0,10	±0,00	±0,04
Mn^2+^	0.03	0.01	0.01	4.88	13.26	52.69	0.06	0.00	0.00
	±0.01	±0.01	±0.02	±8.42	±22.94	±91.25	±0.10	±0.00	±0.00
MoO_4_^2-^	0.04	0.04	0.05	0.49	0.71	0.37	0.01	0.02	0.02
	±0.04	±0.04	±0.08	±0.84	±1.15	±0.59	±0.01	±0.02	±0.02
NH_4_^+^	0.24	0.25	0.23	0.24	0.23	0.19	0.26	0.31	0.10
	±0.41	±0.39	±0.40	±0.34	±0.33	±0.26	±0.37	±0.44	±0.15
NO_3_^–^	4.87	7.29	0.70	53.40	69.50	26.33	48.60	71.88	81.59
	±8.44	±12.63	±1.22	±85.18	±75.85	±27.44	±70.01	±106.71	±136.71
PO_4_^3-^	50.14	61.26	83.54	66.88	89.41	254.00	1.44	2.24	3.38
	±16.79	±44.32	±80.51	±93.54	±90.99	±371.18	±2.50	±3.88	±5.86
SO_4_^2-^	53.58	38.97	36.91	111.68	128.49	138.31	20.41	25.85	17.71
	±31.45	±24.75	±33.29	±158.96	±179.48	±199.45	±24.12	±31.94	±20.16
Zn^2+^	0.06	0.03	0.04	0.74	1.78	6.00	0.04	0.00	0.00
	±0.03	±0.03	±0.04	±1.22	±3.03	±10.36	±0.04	±0.01	±0.00
Total Anion	108.63	107.56	121.20	234.80	290.99	421.90	70.87	100.40	103.14
	±40.83	±77.78	±113.56	±214.11	±206.65	±552.15	±58.03	±90.51	±120.29
Total Cation	3.85	1.55	2.68	33.75	75.97	184.92	0.73	0.54	0.51
	±1.62	±1.11	±2.54	±54.04	±128.86	±317.90	±0.70	±0.51	±0.45
Total Ion	112.48	109.11	123.88	268.55	366.97	606.82	71.60	100.94	103.65
	±41.61	±78.89	±116.10	±267.01	±335.29	±869.52	±58.30	±90.76	±120.47

Sorption / uptake of Ca^2+^, Cu^2+^, Mg^2+^, Mn^2+^ and Zn^2+^ from the micro-nutrient solution by living mycelium and the cell wall extracts at pH 4, 6 and 8 ranged from 2.08 µg (Zn^2+^ and Cu^2+^) to 591.57 (Ca^2+^) (Table 3). For Ca^2+^, Mn^2+^ and Zn^2+^ sorption significantly depended on the pH of the micro-nutrient solution. The living mycelium and the water- and KOH-extracted cell walls absorbed 59%–77%, 74%–88% and 51%–69% of the Ca^2+^, Mn^2+^ and Zn^2+^ from the micro-nutrient solution at pH 8, respectively. This was only 22%–25%, 27%–29% and 8% at pH 4. For Ca^2+^, Mg^2+^ and Zn^2+^ sorption was significantly different between the living mycelium and both cell wall extracts. The living mycelium absorbed 1.3–5.9 times more Zn^2+^ than the KOH-extracted cell walls at pH 4, 6 and 8, while the water-extracted cell walls bound 1.1–8.5 times more Ca^2+^, Mg^2+^ and Zn^2+^ at the different pH values when compared to the KOH-extracted cell walls and living mycelium (Table 3). Together, these data show that metals sorb mainly to the water-extractable part of the cell wall rather than being taken up into the cytoplasm. Moreover, more metals sorb to the cell wall at alkaline pH.

**Table 3:** **Binding of micro-nutrients to the mycelium or to cell walls at different pH values**. Sorption of micro-nutrients from a micro-nutrient solution was determined at pH 4, 6, or 8 using 1 g wet weight living mycelium, 0.24 g wet weight water-extracted cell walls and 0.13 g wet weight KOH-extracted cell walls. The total amount (µg ± sd) of bound micro-nutrient is indicated as well as the percentage of the original amount in the micro-nutrient solution. *n = 2 and not 3.

Micro-nutrient	Living mycelium			dH20-extracted cell walls			KOH-extracted cell walls		
	pH 4	pH 6	pH 8	pH 4	pH 6	pH 8	pH 4	pH 6	pH 8
BO_3_^3-^	7.42	7.74	7.54	8.10	7.92	8.96*	3.07*	1.87*	11.78*
	±3.49	±3.93	±3.90	±4.25	±5.20	±3.80	±2.57	±0.93	±8.78
	18%	19%	18%	20%	19%	22%	7%	5%	29%
Ca^2+^	204.34*	231.72*	537.01*	230.83	423.70	591.57	64.82	50.63*	410.79
	±112.91	±107.21	±14.96	±127.63	±437.11	±386.86	±111.69	±70.82	±168.00
	25%	29%	67%	29%	53%	74%	8%	6%	51%
Cu^2+^	6.53*	7.13*	7.14*	8.51	17.29	17.59	2.80*	7.62*	12.37
	±0.22	±0.78	±0.80	±2.87	±13.43	±13.81	±3.39	±6.18	±4.50
	41%	45%	45%	54%	109%	111%	18%	48%	78%
Mg^2+^	165.69	178.98	235.53	194.75	375.56	396.79	58.52	45.46*	209.83
	±84.23	±84.61	±75.36	±118.13	±416.95	±391.93	±101.20	±64.30	±130.50
	23%	25%	32%	27%	52%	54%	8%	6%	29%
Mn^2+^	84.56	96.22	227.15	104.60	200.41	338.62	30.30	23.48*	263.47
	±25.81	±21.03	±134.59	±61.31	±211.74	±198.77	±51.93	±33.21	±122.13
	22%	25%	59%	27%	52%	88%	8%	6%	69%
MoO_4_^2-^	10.38	4.59*	4.60	7.99*	9.43	8.09*	6.96*	1.08*	7.03*
	±6.03	±1.94	±2.11	±3.17	±10.05	±9.54	±3.47	±1.46	±3.47
	50%	22%	22%	39%	46%	39%	34%	5%	34%
NO_3_^–^	1635.63	1824.46	1669.79	1686.80	2771.32	2770.59	2950.16	2053.05	2359.15
	±811.43	±1250.75	±815.17	±1725.98	±1452.27	±1885.25	±1197.09	±2154.57	±1423.06
	29%	33%	30%	30%	50%	50%	53%	37%	42%
PO_4_^3-^	446.95	475.67	1190.34	404.43	312.56	949.21	215.00*	103.83*	1102.23
	±202.29	±220.24	±646.12	±133.61	±184.28	±547.95	±297.94	±137.34	±661.72
	19%	20%	50%	17%	13%	40%	9%	4%	46%
SO_4_^2-^	600.44	615.69	610.58	647.57	487.47	473.94	288.33	238.96*	786.48
	±310.28	±350.65	±380.44	±527.46	±531.71	±492.10	±447.20	±250.53	±723.08
	20%	21%	21%	22%	17%	16%	10%	8%	27%
Zn^2+^	7.64	12.29	25.08	8.93	15.94	27.77	2.57	2.08*	19.99
	±2.57	±4.04	±13.19	±4.99	±11.49	±14.97	±4.34	±2.92	±9.34
	23%	38%	77%	27%	49%	85%	8%	6%	61%
Total Anion	2700.81	2928.15	3482.86	2754.89	3588.71	4207.81	3388.50	2283.54	4260.40
	±525.28	±1058.70	±615.58	±2145.14	±2049.82	±1754.01	±1608.88	±2115.43	±999.91
	25%	27%	32%	25%	33%	38%	31%	21%	39%
Total Cation	398.48	446.72	850.53	547.62	1032.91	1372.34	158.07	86.18	916.45
	±230.20	±235.52	±461.71	±313.93	±1089.63	±970.82	±272.04	±138.52	±361.49
	20%	23%	43%	28%	53%	70%	8%	4%	47%
Total Ion	3099.29	3374.87	4333.39	3302.51	4621.62	5580.15	3546,58	2369,72	5176,85
	±334.95	±869.30	±846.34	±2260.06	±2603.00	±2137.84	±1823.11	±2134.87	±937.09
	19%	21%	27%	21%	29%	35%	22%	15%	32%

Next, binding of anions was analyzed. Binding of BO_3_^3-^, MoO_4_^2-^, PO_4_^3-^, SO_4_^2-^ and NO_3_^–^ ranged between 1.08 µg (MoO_4_^2-^) to 2.95 mg (NO_3_^–^) by the living mycelium, or water-extracted or KOH-extracted cell walls when incubated at pH 4, 6 and 8 (Table 3). Only binding of PO_4_^3-^ was significantly higher at pH 8 than at pH 4 or 6. The living mycelium and the water- and KOH-extracted cell walls took up 40% −50% of the PO_4_^3-^ at pH 8, but this was only 9%–19% at pH 4. Binding of BO_3_^3-^, MoO_4_^2-^, PO_4_^3-^, SO_4_^2-^ and NO_3_^–^ was similar for the living mycelium and the water- and KOH-extracted cell walls. Together, these data show that anions bind to cell walls of *S. commune*, which in most cases is independent of pH.

Living mycelium and water- and KOH-extracted cell walls were incubated with an anion solution (Table 1) to see if binding of the anions (BO_3_^3-^, MoO_4_^2-^, NO_3_^–^, PO_4_^3-^ and SO_4_^2^) to the cell wall depended on the metal ions in the micro-nutrient solution (Table 3, Table 4). The living mycelium took up 1.05 (BO_3_^3-^ at pH 6) to 2.15 (NO_3_^–^ at pH 4) fold more BO_3_^3-^ or NO_3_^–^ from the micro-nutrient than from the anion solution at pH 4, 6 or 8. The living mycelium also took up 2.45 times more PO4^3-^ from the micro-nutrient solution at pH 8, but 0.93 and 0.97 times less at pH 4 and pH 6 respectively. Moreover, it absorbed 1.04 and 1.02 times more SO_4_^2-^ from the anion than from the micro-nutrient solution at pH 6 or 8, respectively. Next, the water-extracted cell wall bound between 1.31 (PO_4_^3-^ at pH 8) to 2.86 (PO_4_^3-^ or SO_4_^2-^ at pH 6) times more BO_3_^3-^, PO_4_^3-^ or SO_4_^2-^ from the anion than from the micro-nutrient solution at pH 4, 6 or 8. Similarly, 1.75 times more NO_3_^–^ was absorbed at pH 4, however 0.74 and 0.94 times less binding was found at pH 6 and 8. The KOH-extracted cell walls took up 2.11 (SO_4_^2-^) to 3.17 (PO_4_^3-^) more BO_3_^3-^, PO_4_^3-^ and SO_4_^2-^ from the micro-nutrient solution than from the anion solution at pH 8. However, 0.91 (PO_4_^3-^ at pH 4) to 0.49 (BO_3_^3-^ at pH 4) times less of these anions were absorbed at pH 4 and pH 6. The KOH-extracted cell walls took up 1.21 and 1.22 times more NO_3_^–^ from the anion than from the micro-nutrient solution at pH 6 and pH 8 respectively, yet 1.10 times less at pH 4. These results indicate that sorption of BO_3_^3-^, PO_4_^3-^ and NO_3_^–^ by living mycelium, NO_3_^–^ by water-extracted cell walls and all anions by KOH-extracted cell walls can be mediated in part by metal ions.

**Table 4:** **Binding of anions to the mycelium or to cell walls at different pH values**. Sorption of anions from an anion solution was determined at pH 4, 6, or 8 using 1 g wet weight living mycelium, 0.24 g wet weight water-extracted cell walls and 0.13 g wet weight KOH-extracted cell walls. The total amount (µg ± sd) of bound micro-nutrient is indicated as well as the percentage of the original amount in the micro-nutrient solution. *n = 2 and not 3.

Micro-nutrient	Living mycelium			dH20-extracted cell walls			KOH-extracted cell walls		
	pH 4	pH 6	pH 8	pH 4	pH 6	pH 8	pH 4	pH 6	pH 8
BO_3_^3-^	6.46	7.36	7.02	14.70	16.31	15.34	4.15*	3.83*	3.75*
	±2.68	±3.08	±3.43	±11.51	±13.94	±14.40	±4.10	±3.70	±3.42
	16%	18%	17%	36%	40%	37%	10%	9%	9%
MoO_4_^2-^	0.04	0.03	0.08*	0.74	0.69	0.52	0.01	0.03	0.05*
	±0.02	±0.03	±0.08	±1.00	±1.15	±0.73	±0.01	±0.01	±0.02
	0.2%	0.1%	0.4%	4%	3%	3%	0.1%	0.1%	0.3%
NH_4_^+^	499.90	1304.62	1406.44	962.26	854.78	934.05	1021.26*	771.62*	1046.64*
	±865.86	±848.77	±341.62	±51.84	±422.47	±351.70	±698.00	±219.93	±841.31
	16%	41%	44%	30%	27%	29%	32%	24%	33%
NO_3_^–^	652.13	1104.12	1396.86	2955.42	2618.00	2037.49	2671.97	2482.66	2888.02
	±714.34	±445.45	±29.02	±2434.99	±2482.38	±2147.73	±2831.82	±2578.12	±1900.38
	12%	20%	25%	53%	47%	37%	48%	44%	52%
PO_4_^3-^	481.62	491.61	485.02	991.94	1133.75	1240.21	236.16	210.49	297.38*
	±157.76	±243.33	±294.09	±695.90	±987.13	±1302.88	±365.22	±342.43	±406.22
	20%	21%	20%	42%	48%	52%	10%	9%	13%
SO_4_^2-^	599.42	638.69	623.25	1125.77	1393.82	1119.55	474.81	427.06	372.73
	±239.47	±325.72	±408.42	±787.56	±976.11	±990.19	±636.00	±587.32	±599.99
	20%	22%	21%	38%	47%	38%	16%	14%	13%
Total Anion	1739.67	2241.79	2512.20	5088.33	5162.58	4412.93	3385.73	3122.79	3461.54
	±330.34	±271.63	±681.41	±3300.58	±3777.66	±3486.58	±3818.77	±3476.32	±2536.70
	16%	20%	23%	46%	47%	40%	31%	28%	32%
Total Ion	2239.58	3546.41	3918.64	6050.59	6017.36	5346.98	4066.57	3637.20	4159.30
	±1175,58	±978.32	±540.72	±3260.43	±3986.52	±3781.81	±4476.54	±3759.38	±3119.63
	16%	25%	28%	43%	43%	38%	29%	26%	29%

The living mycelium, and the water- and KOH-extracted cell walls loaded with micro-nutrients at pH 4, 6 or 8 from the micro-nutrient (Table 5) or anion solution (Table 6) were washed at pH 8 and incubated in 0.1 mM HCl (pH 4) to release the micro-nutrients. Living mycelium and cell walls that had been incubated in water at pH 4–8 were also washed and incubated in HCl as a control (data not shown). Release of the absorbed Ca^2+^, Cu^2+^, Mg^2+^, Mn^2+^ and Zn^2+^ from the living mycelium or water- or KOH-extracted cell walls ranged between 6% (Mg^2+^) to 103% (Cu^2+^) (Table 5). A total of 26% to 48% of the absorbed Ca^2+^, Mg^2+^, Mn^2+^ and Zn^2+^ was released from the living mycelium that had bound micro-nutrients at pH 8, while this was only 11%−18% after absorption at pH 4 (Table 5). The water-extracted cell walls released 22% to 46% of the Ca^2+^, Mg^2+^, Mn^2+^ and Zn^2+^ after absorption at pH 8, while this was 6% to 10% after absorption at pH 4. In contrast, the KOH-extracted cell walls released 22% to 27% of the Ca^2+^, Mn^2+^ and Zn^2+^ that had bound at pH 8, but 47% to 61% of those that had bound at pH 4. Together, these data show that metals are released from the cell wall under acidic conditions.

**Table 5:** **Release of micro-nutrients from micro-nutrient loaded mycelium or cell walls at pH 4.** A total of 1 g wet weight living mycelium, 0.24 g wet weight water-extracted cell walls and 0.13 g wet weight KOH extracted cell walls were loaded with micro-nutrients from a micro-nutrient solution at pH 4, 6, or 8. Release of the micro-nutrients was measured after 30 min incubation in 0.1 mM HCl (pH 4). The total amount (µg ± sd) of released micro-nutrient is indicated as well as the percentage of the original amount that was bound to the cell wall. *n = 2 and not 3.

Micro-nutrient	Living mycelium			dH20-extracted cell walls			KOH-extracted cell walls		
	pH 4	pH 6	pH 8	pH 4	pH 6	pH 8	pH 4	pH 6	pH 8
BO_3_^3-^	2.68*	5.30*	6,35*	2.03	4.92	5.25	1.35*	0.69	3.76
	±0.10	±4.11	±3.70	±2.01	±6.36	±5.75	±0.64	±0.64	±6.28
	36%	69%	84%	25%	62%	59%	44%	37%	32%
Ca^2+^	26.26	35.73	153.69	18.54	59.74	219.22	30.31	17.44*	112.87
	±43.97	±56.42	±250.14	±29.89	±50.26	±247.47	±26.75	±21.01	±127.29
	13%	15%	29%	8%	14%	37%	47%	34%	27%
Cu^2+^	3.23	3.57	3.80	2.29	8.50	8.22	2.89	7.04	3.56
	±4.99	±5.43	±5.82	±3.50	±6.94	±6.81	±3.03	±5.44	±2.59
	49%	50%	53%	27%	49%	47%	103%	92%	29%
Mg^2+^	18.43	21.91	60.51	12.48	41.48	85.94	26.49	37.19	97.00
	±31.53	±35.97	±98.86	±20.76	±36.47	±83.24	±23.33	±41.16	±134.15
	11%	12%	26%	6%	11%	22%	45%	82%	46%
Mn^2+^	15.08	19.50	108.13	10.67	31.16	151.06	15.43	10.04*	57.94
	±25.34	±32.64	±175.86	±17.29	±25.47	±183.21	±13.69	±12.27	±57.25
	18%	20%	48%	10%	16%	45%	51%	43%	22%
MoO_4_^2-^	1.50	0.31	0.41	2.42	2.09	1.97	2.11	0.59	2.40
	±2.56	±0.52	±0.66	±3.26	±2.52	±2.17	±1.66	±0.56	±3.75
	15%	7%	9%	30%	22%	24%	30%	54%	34%
NO_3_^–^	180.38	143.53	1047.85	54.92	24.02	137.20	65.77	78.07	59.58
	±214.92	±105.65	±1466.40	±83.03	±30.94	±121.24	±66.41	±83.05	±56.93
	11%	8%	63%	3%	1%	5%	2%	4%	3%
PO_4_^3-^	106.56*	137.82*	465.40	68.19*	159.90*	883.26*	90.82*	52.13*	388.06
	±53.36	±8.41	±679.87	±23.82	±63.97	±713.43	±30.18	±50.00	±290.17
	24%	29%	39%	17%	51%	93%	42%	50%	35%
SO_4_^2-^	119.58	148.97	228.22	119.29*	60.26*	117.77*	133.39	59.97	490.14*
	±124.68	±128.55	±207.16	±149.09	±59.63	±146.85	±113.51	±49.86	±637.68
	20%	24%	37%	18%	12%	25%	46%	25%	62%
Zn^2+^	0.91	3.25	9.77	0.68	4.23	12.74	1.57	1.75*	4.73
	±1.52	±5.47	±15.82	±0.97	±3.43	±14.93	±1.68	±2.20	±4.63
	12%	26%	39%	8%	27%	46%	61%	84%	24%
Total Anion	374.29	388.22	1746.11	183.55	177.80	811.78	262.70	157.70	651.21
	±323.22	±303.19	±2319.28	±137.42	±143.90	±900,41	±235.24	±125.63	±8910.09
	14%	13%	50%	7%	5%	19%	8%	7%	15%
Total Cation	63.90	83.95	335.90	44.65	145.29	477.37	76.70	63.72	276.11
	±107.33	±135.89	±546.45	±72.40	±121.60	±532.28	±68.27	±49.15	±323.06
	16%	19%	39%	8%	14%	35%	49%	74%	30%
Total Ion	438.19	472.17	2082.01	227.21	322.90	1288.98	339.40	220.42	927.32
	±376.44	±362.89	±2859.68	±206.61	±262.49	±1424.37	±302.16	±171.00	±1211.93
	14%	14%	48%	7%	7%	23%	10%	9%	18%

**Table 6:** **Release of anions from anion loaded mycelium or cell walls at pH 4.** A total of 1 g wet weight living mycelium, 0.24 g wet weight water-extracted cell walls and 0.13 g wet weight KOH extracted cell walls were loaded with anions from an anion solution at pH 4, 6, or 8. Release of the micro-nutrients was measured after 30 min incubation in 0.1 mM HCl (pH 4). The total amount (µg ± sd) of released anions is indicated as well as the percentage of the original amount that was bound to the cell wall. *n = 2 and not 3.

Micro-nutrient	Living mycelium			dH20-extracted cell walls			KOH-extracted cell walls		
	pH 4	pH 6	pH 8	pH 4	pH 6	pH 8	pH 4	pH 6	pH 8
BO_3_^3-^	1.99*	1.19*	1.03*	9.80	1.34	1.87	0.97	0.71	0.64
	±2.43	±1.76	±1.44	±14.92	±1.18	±1.52	±0.85	±0.69	±0.81
	31%	16%	15%	67%	8%	12%	23%	19%	17%
MoO_4_^2-^	0.01	0.00	0.00	0.02	0.00	0.01	0,00*	0,00*	0,00*
	±0.02	±0.03	±0.00	±1.00	±0.01	±0.02	±0.01	±0.01	±0.02
	39%	0%	5%	2%	0%	1%	31%	0%	0%
NH_4_^+^	32.82	101.55	88.44	0,00	0,00	0,00	0,00	0,00	0,00
	±29.80	±145.95	±118.73	±0,00	±0,00	±0,00	±0,00	±0,00	±2.99
	7%	8%	6%	0%	0%	0%	0%	0%	0%
NO_3_^–^	141.31	176.49	236.43	79.92	0,08	70.73	123.73	113.50	183.32
	±123.55	±86.45	±123.02	±69.07	±0.14	±104.10	±99.46	±31.05	±235.90
	22%	16%	17%	3%	0%	3%	5%	5%	6%
PO_4_^3-^	90.27	80.46	53.78	222.97	39.37*	75.25*	36.75	52.75	26.08
	±113.98	±93.03	±75.72	±252.54	±50.84	±103.55	±31.70	±45.27	±38.43
	19%	16%	11%	22%	3%	6%	16%	25%	9%
SO_4_^2-^	160.70	97,27	66,09	715,77	19,38*	100,70*	55,84*	80,99	115,07*
	±257.47	±133.77	±103.24	±953.87	±19.61	±125.46	±70.23	±67.87	±599.99
	27%	15%	11%	64%	1%	9%	12%	19%	31%
Total Anion	394.62	355.43	278.20	1028.15	60.09	189.91	198.69	248.97	248.42
	±449.00	±238.36	±244.80	±1154.86	±60.60	±295.00	±78.67	±138.58	±200.76
	23%	16%	11%	20%	1%	4%	6%	8%	7%
Total Ion	426.44	456.98	366.65	1028.15	40.59	189.91	198.69	247.97	249.83
	±425.37	±259.44	±281.23	±1154.86	±60.60	±295.00	±78.67	±138.58	±202.95
	19%	13%	9%	17%	1%	4%	5%	7%	6%

Release of BO_3_^3-^, MoO_4_^2-^, NO_3_^–^ or SO_4_^2-^ from the living mycelium and the water- and KOH-extracted cell wall extracts did not depend on the pH at which the ions had been sorbed from the micronutrient solution (Table 5). However, desorption of PO_4_^3-^ from the living mycelium and water-extracted cell walls did depend on the pH at which this anion was sorbed (Table 5). 39%–93% of the bound PO_4_^3-^ was released after absorption at pH 8, while this was only 17%–24% after absorption at pH 4. These data show that anions are released from the cell wall at acidic pH. The release of anions from living mycelium and cell walls that had been incubated in micro-nutrient and anion solutions were compared to see if the presence of metals affected the desorption of anions. After sorption at pH 8 and desorption at pH 4, the living mycelium released 84% and 37% of the BO_3_^3-^ or SO_4_^2-^, respectively, that had sorbed from the micro-nutrient solution (Table 5), while only 15% and 11% of these anions were released after sorption from the anion solution (Table 6). The water- and KOH-extracted cell walls released 59% and 25% or 32% and 62%, respectively, of the BO_3_^3-^ and SO_4_^2-^ that were bound from the micro-nutrient solution (Table 5), while 12% and 9% or 17% and 31% were released from those absorbed from the anion solution (Table 6). Together, these results show that the presence of metals increases the desorption of anions from the cell wall polymers.

### 3.2 Binding sites of ions to the *S. commune* cell wall

**Fig. 1:**
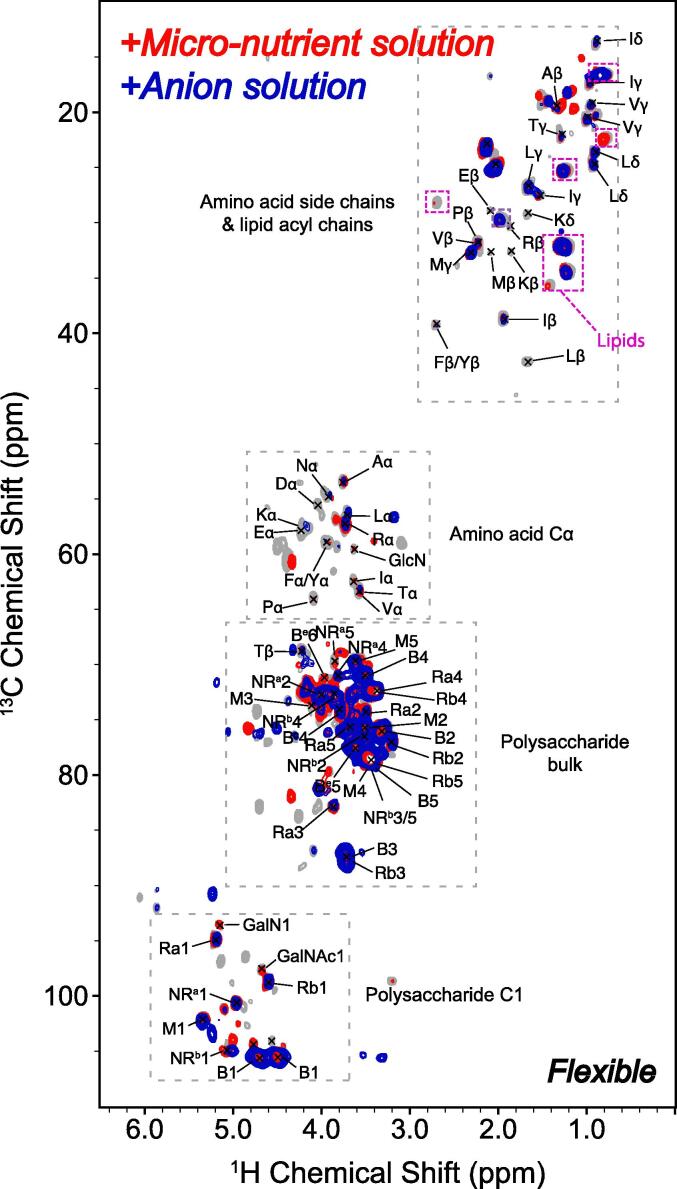
**^1^H–^13^C correlation spectra of the flexible layer of the *S*. *commune* H4-8A cell wall before (grey) and after (red) sorption of micro-nutrients from a micro-nutrient solution at pH5.5.** Signals modulated after incubation in the anion solution as well as in the micro-nutrient solution are indicated in blue. Abbreviations are used to denote the following polysaccharides: β-(1,3)-glucan (B), non-reducing end β-(1,3)-glucan (NR^b^), reducing end β-(1,3)-glucan (Rb), α-(1,3)-glucan (A), chitin (Ch); mannan (M), non-reducing end α-glucan (NR^a^), reducing end α-glucan (Ra), and β-(1,6)-glucan (B^e^).

Micro-nutrients from the micro-nutrient solution were sorbed to ^13^C and ^15^N-labeled water-extracted cell walls at pH 5.5 and analyzed by ssNMR to determine their binding sites (Fig. 1). Signals belonging to rigid components were not sensitive to the addition of micro-nutrients. In contrast, signals of flexible amino acid carbon backbone and side-chains were heavily modulated after sorption of the micro-nutrients suggesting ionic binding to the mobile cell wall proteins. This effect was particularly strong for the charged amino acids lysine, arginine, and glutamic acid but also signals from the aromatic amino acids phenylalanine and tyrosine were perturbed. The side chains of hydrophobic amino acids isoleucine and valine remained largely unaffected and therefore likely do not interact at the micro-nutrient concentrations that were used. Furthermore, low intensity signals around the polysaccharide bulk region and the C1 region disappeared but the main signals were not affected. Together, data indicate that micro-nutrients bind to proteins as well as to lowly abundant mobile polysaccharides in the cell wall.

Water-extracted cell wall material was also incubated with the anion solution at pH 5.5. We found that the same mobile signals were modulated for the anion- and the micro-nutrient solution, as is visible in the overlay in Fig. 1 (blue). This indicates that the same cell wall moieties bind both metals and anions. Again, the dipolar-based spectra did not demonstrate any observable binding events of anions to rigid cell wall components.

## 4 Discussion

Living mycelium and water- and KOH-extracted cell walls were exposed to micro-nutrients. The concentration of the nutrients in these solutions were based on the Hoagland solution that is used to grow plants. Results show that cations (i.e. metals) and anions mainly sorb to the cell wall. The metals had a higher affinity for water-extractable cell walls than for those extracted with KOH and sorption was higher at alkaline pH. On the other hand, sorption of anions was similar in the case of water- and KOH-extracted cell walls and was not affected by pH with the exception of phosphate. Results also indicate that anions sorb directly to cell wall polymers as well as to metals that are bound to the cell wall.

Binding of metals, especially at high concentration, to the cell wall is believed to be more important than active uptake into the cytoplasm to remove metals from solution (Abbas et al., 2014). This was confirmed in our current study. Water-extracted cell walls sorbed 1.10–2.10 times more Ca^2+^, Mg^2+^ and Zn^2+^ at the different pH values when compared to living mycelium. A lower sorption by water-extracted cell walls would have been expected if uptake into the cytosol would have been a main contributor of clearance of metals from the solution.

Fungal hyphae can sorb metals by complexation, coordination, chelation, ion exchange, adsorption, and microprecipitation (Remacle, 1990, Naja and Volesky, 2011, Abbas et al., 2014). For instance, metals can precipitate, either or not forming crystals, as carbonates, oxides, oxalates, sulfates or phosphates on the cell surface or in the inter-cell wall spaces (Naja and Volesky, 2011; Remacle, 1990). They can also sorb to the cell wall by ion-exchange, complexation and coordination with carboxyl or phosphate groups in proteins and uronic acids, and nitrogen rich ligands in proteins, chitin and chitosan (Abbas et al., 2014, Naja and Volesky, 2011). Moreover, C5 of (1,3)-(1,6)-β-glucan can bind Cu^2+^ (Safeer et al., 2023). The fungal cell wall has a net negative charge (Remacle, 1990) but more metals can bind to the cell wall at alkaline pH because of an increase in free ionizable sites (Abbas et al., 2014). Indeed, binding of Ca^2+^, Mn^2+^ and Zn^2+^ to the cell wall of *S. commune* was higher at pH 8 than at pH 4 or 6. Absorption of anions by fungal hyphae is less well-defined. The amine and amide sites of chitin and chitosan in the KOH-extractable part of the cell wall can be protonated to bind anion species. However, this only explains the binding of anions at pH levels below the pKa of < 3.5 for chitin, or 6.5 for chitosan (Naja and Volesky, 2011).

No indications have been found for binding of Cu^2+^ to chitin, (1,3)-α-glucan and (1,3)-β-glucan representing the main polysaccharides in the cell wall of *S. commune* (Safeer et al., 2023). Instead, Cu^2+^, at a concentration of 0.74 mM, binds to mobile cell wall proteins and to lowly abundant mobile polysaccharide species. Only at 18.5 mM, Cu^2+^ also binds to the C5 of rigid (1,3)(1,6)-β-glucan. We here showed that other micronutrients (cations and / or anions) also bind to the mobile proteins and the lowly abundant mobile polysaccharide species. No indications were found for binding to the C5 of rigid (1,3)(1,6)-β-glucan, which may be explained by the fact that the living mycelium and the extracted cell walls were exposed to relatively low concentrations of anions and cations (e.g concentration of Mn^2+^, Ca^2+^, and Mg^2+^ ranged between 0.8 and 3 mM). We do not know which molecules from the micronutrient and anion solutions exactly bind to the mobile proteins and lowly abundant mobile polysaccharides. This should be assessed in future studies. Based on the NMR spectra, we do know that there are no other main binding sites for anions and cations in the cell wall at the concentrations that were used. Of interest, cell wall composition depends on culture conditions (Gow et al., 2017). Therefore, it would be interesting to assess how the cell wall composition is affected by the pH of the medium and, in turn, how this would affect micro-nutrient binding.

This study showed that sorption of BO_3_^3-^, MoO_4_^2-^, PO_4_^3-^, SO_4_^2-^ and NO^3–^ from the micro-nutrient and anion solutions by living mycelium and water- and KOH-extracted cell walls ranged between 0 µg and 2.95 mg. This difference can be explained, at least in part, by the different amounts of these anions in the micro-nutrient and anion solutions. In contrast to the other anions, sorption of PO_4_^3-^ was higher at alkaline than at acidic pH environment. This can be explained by the ability of this anion to form complexes with metal ions at this pH. Notably, our results indicate that sorption of BO_3_^3-^, PO_4_^3-^ and NO_3_^–^ by living mycelium, NO_3_^–^ by water-extracted cell walls and all anions by KOH-extracted cell walls is mediated, at least in part, by metal ions. This is concluded from the fact that more of these anions were sorbed in the presence of metal ions. As mentioned above, our NMR data show that the anions in the micro-nutrient solution bind at the same positions that were previously reported for the cation Cu^2+^ at pH 5.5 (Safeer et al., 2023). This could be mediated by the binding of the metal ions.

This study showed that BO_3_^3-^, Ca^2+^, Cu^2+^, Mg^2+^, Mn^2+^, MoO_4_^2-^, PO_4_^3-^, SO_4_^3-^, Zn^2+^ and NO_3_^–^ can be desorbed from the cell wall at low pH (i.e. pH 4). The fact that more anions were released when sorption had been done in the presence of metal ions implies that these metal ions not only stimulates binding but also increases their release at low pH. The metals in the cell wall may be exchanged for protons at low pH (Abbas et al., 2014, Naja and Volesky, 2011), thereby resulting in the release. Consequently, the anions would also be released.

Together, we here showed that the fungal cell wall of *S. commune* can bind a wide variety of micro-nutrients. This binding may have different functions. First, it may not only act as a storage for Cu^2+^ (Probst et al., 2022) but also for other micro-nutrients. These micro-nutrients can be taken up by the cytoplasm when binding to the cell wall is released, for instance due to lowering of the environmental pH. Also by sequestering micro-nutrients to the cell wall, the concentration in the environment is reduced, thereby depriving other microbes from these essential nutrients (Probst et al., 2022). On the other hand, binding of metals to the cell wall can protect against toxic influx when environmental concentrations are high.

## Funding

This work was funded by the Dutch Research Council (NWO) domain Applied and Engineering Sciences (Project number 18425) as well as the National Roadmap Large-Scale NMR Facility of the Netherlands (NWO grant 184.035.002).

## CRediT authorship contribution statement

**Fleur E.L. Kleijburg:** Writing – original draft, Methodology, Investigation, Formal analysis, Conceptualization. **Adil A. Safeer:** Writing – original draft, Methodology, Investigation, Formal analysis. **Marc Baldus:** Writing – review & editing, Supervision, Methodology, Funding acquisition. **Han A.B. Wösten:** Writing – review & editing, Supervision, Funding acquisition, Conceptualization.

## Declaration of Competing Interest

The authors declare that they have no known competing financial interests or personal relationships that could have appeared to influence the work reported in this paper.
